# Effective pore size and radius of capture for K^+^ ions in K-channels

**DOI:** 10.1038/srep19893

**Published:** 2016-02-02

**Authors:** Hans Moldenhauer, Ignacio Díaz-Franulic, Fernando González-Nilo, David Naranjo

**Affiliations:** 1 Centro Interdisciplinario de Neurociencia de Valparaíso, Universidad de Valparaíso, Playa Ancha Valparaíso, Chile; 2 Universidad Andrés Bello, Center for Bioinformatics and Integrative Biology, Santiago, Chile and Fundación Fraunhofer-Chile, Las Condes, Chile

## Abstract

Reconciling protein functional data with crystal structure is arduous because rare conformations or crystallization artifacts occur. Here we present a tool to validate the dimensions of open pore structures of potassium-selective ion channels. We used freely available algorithms to calculate the molecular contour of the pore to determine the effective internal pore radius (*r*_E_) in several K-channel crystal structures. *r*_E_ was operationally defined as the radius of the biggest sphere able to enter the pore from the cytosolic side. We obtained consistent *r*_E_ estimates for MthK and Kv1.2/2.1 structures, with *r*_E_ = 5.3–5.9 Å and *r*_E_ = 4.5–5.2 Å, respectively. We compared these structural estimates with functional assessments of the internal mouth radii of capture (*r*_C_) for two electrophysiological counterparts, the large conductance calcium activated K-channel (*r*_C_ = 2.2 Å) and the Shaker Kv-channel (*r*_C_ = 0.8 Å), for MthK and Kv1.2/2.1 structures, respectively. Calculating the difference between *r*_E_ and *r*_C_, produced consistent size radii of 3.1–3.7 Å and 3.6–4.4 Å for hydrated K^+^ ions. These hydrated K^+^ estimates harmonize with others obtained with diverse experimental and theoretical methods. Thus, these findings validate MthK and the Kv1.2/2.1 structures as templates for open BK and Kv-channels, respectively.

K-channel crystal structures reveal that they are machines optimized for efficient and selective K^+^ ion transport. Thanks to the groundbreaking work from the Mackinnon lab, we know the structure of several K-channels in detail^1^. A great deal of effort has been invested in building a conceptual scaffold that makes functional sense of these K-channel structures. In the pore domain of K-channels, this scaffold works pretty well for understanding toxin binding and K^+^ selectivity. This is partly because the external vestibule and the selectivity filters are experimentally more accessible, and also because of the low variance in atom’s space coordinates among different K-channels across diverse crystallization conditions^2^,^3^,^4^,^5^,^6^. Thus, the structure of the external vestibule together with that of the selectivity filter enjoy a solid functional reputation. By contrast, the internal vestibule of the pore seems to be much less well-defined. On one hand, the dimensions of the cytosolic aspects of the pore structure differ from channel to channel in the various crystal structures^3^,^5^,^7^,^8^. On the other, the internal vestibule seems to be the flexible part of the protein where the voltage controlled gate is located. For example, the structures of KcsA (PDB:1K4C) and Slo2.2 (PDB:5A6E) seem to correspond to channels crystallized in the closed conformation because their pore’s internal opening size is smaller than that of a hydrated K^+^ 5,9. Meanwhile, the structures of the bacterial MthK (PDB: 4HYO) and of the mammalian Kv1.2/2.1 paddle chimera (PDB: 2R9R) appear to be those of open channels, with the MthK internal vestibule being ~10 Å wider than that of Kv1.2/2.1^3^,^7^,^10^,^11^.

The size difference in the pore internal vestibule in the structure of the MthK vs. the Kv1.2/2.1 chimera structure was somehow electrophysiologically corroborated in their functional counterparts, the mouse large conductance calcium and voltage gated K^+^-channel (BK) and the *Drosophila* voltage gated Shaker K^+^-channel, respectively. Cysteine substitution scanning accessibility experiments^12^,^13^ in Shaker and BK channels, and also side-chain volume changes in residues located at the internal entrance of BK^14^, suggested a pore several angstrom wider than Shaker’s. Nevertheless in diffusional determinations of their radii of capture, BK is only ~1.4 Å wider (see below)^15^,^16^. To what extent do these differences represent structurally different K-channels, or do they just reflect diverse conformations? Are they the result of rarely visited conformational states or are they caused by crystallization artifacts? These questions are especially important when, based on sequence homology or functional properties such as single channel conductance and pharmacology, we use the atomic coordinates of one channel as a structural template for a distantly related one, as is the case when using the structure of MthK to model the BK channel.

Here, we present a simple methodology to estimate the functional dimensions of the internal entrance to the pore in K^+^-channel structural models. Briefly: for several K^+^-channel crystal structures we first estimated their effective pore size^17^ and then compared these values with functional estimations of their diffusional radius of capture obtained from diffusion limited unitary currents^15^,^16^,^18^,^19^,^20^. To validate the methodology, the difference between these two estimates must equal robust approximations of the hydrodynamic radius of K^+^ ions.

## The Effective Pore Size

The effective dimension for permeation in a K^+^-channel pore is determined by the effective size of the K^+^ ion, which, in turn, is determined by the average number of water molecules forming its hydration shell. At one extreme, if K^+^ ion coming from the cytosolic side were a point charge, having no physical dimension and no hydration shell, and the protein were a rigid structure, the effective sectional area to pass should be delimited by the Van der Waals envelope of the pore entrance^21^. But if instead K^+^ were a solid spherical body of finite dimensions, it will not pass across surface tortuosities smaller than its own size, resulting in a less rugose, and decreased, sectional area available for permeation. In this other extreme, if the hydrated K^+^-complex is larger than the pore entrance, it will not enter. In this latter case, from the ion’s perspective, the pore is closed. Thus, if we assume the ion to be a rigid spherical probe of variable size moving into a circular pore, we define the effective pore radius (*r*_E_) as the radius of the largest sphere that is able to pass through the pore^17^.

**Figure 1:**
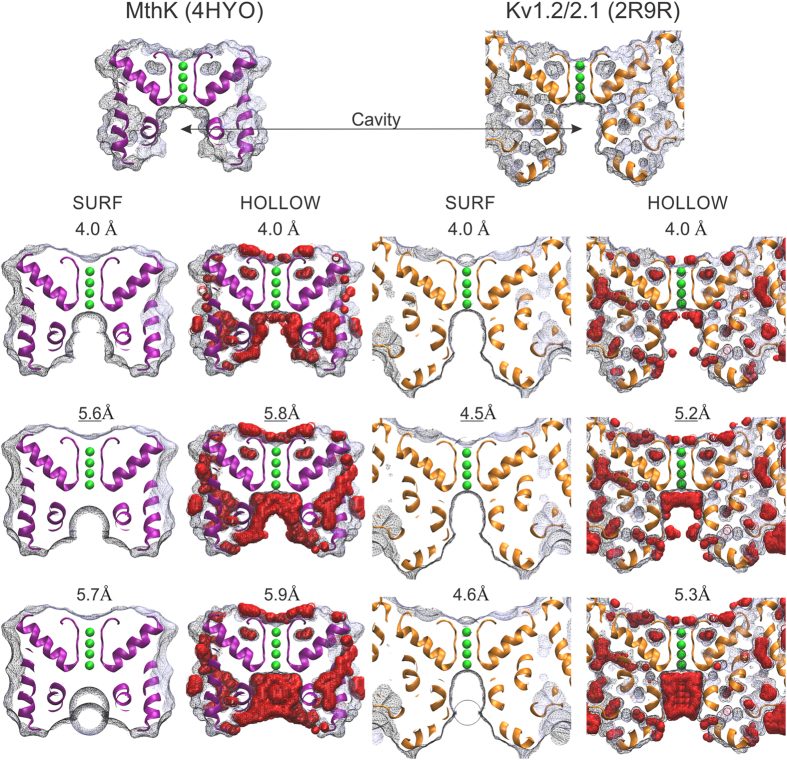
The effective opening radius in two K-channel structures. Shown are ~5 Å slabs of the molecular surface of the large conductance bacterial channel MthK (PDB: 4HYO) and the chimeric Kv1.2/2.1 voltage gated K-channel (PDB: 2R9R). The molecular surfaces were calculated as indicated in the main text. The probe radius (in Å) is shown above each representation. The top row shows the molecular surfaces for a 4 Å-radius probe (about the size of a hydrated K^+^). The middle row (with underlined radii values) shows the size of the largest spheres able to pass through the pore (r_E_). The lower row shows that probes 0.1 Å bigger cannot pass. With SURF, these probes leave a dimple or a bump at the pore entrance, depending on whether the rolling trajectory began outside or inside the cavity, respectively (radius = 5.7 Å and 4.6 Å, for MthK and Kv1.2/2.1, respectively). Meanwhile, with HOLLOW, the non-permeating probes (radius = 5.9 Å and 5.3 Å, for MthK and Kv1.2/2.1, respectively) leave the cavity full of virtual O atoms (red spheres). Two opposite pore-helices and K^+^ ions in selectivity filter (in green) are shown for reference. The T1, the β-subunit, and the voltage sensing domains are not represented in 2R9R for clarity. Figure prepared with VMD (http://www.ks.uiuc.edu/Research/vmd/).

To estimate *r*_E_ on potassium channel structures we used freely available algorithms (SURF, MSMS, and HOLLOW). These routines calculate the molecular surface of proteins left by the contact of rigid spherical test probes of variable radius. In SURF and MSMS the probe is rolled on top of the Van der Waals surface of the channel structure^21^,^22^,^23^ (Fig. 1 in^21^). When the probe contacts simultaneously more than one atom, these contacts produce a series of disconnected patches that are filled with concave surfaces having the probe´s curvature. Thus, the resulting envelope is a molecular surface that is less detailed as the probe grows bigger (See for example first column in Fig. 1). On the other hand, HOLLOW consisted in filling the protein holes and voids with virtual overlapping oxygen atoms placed at fixed intervals defined in a 0.2–0.5 Å cubic grid^24^. The molecular surface is then defined by the envelope of the virtual oxygen atoms that are not contacted by the probe (See for example second column in Fig. 1).

Examples of the molecular surfaces left by SURF and HOLLOW (Fig. 1) show that smaller probes go inside the cavity defining a detailed molecular surface of the pore walls, whereas bigger probes leave poorer surface detail. Probes big enough will not be able to enter, transforming the pore into another dimple in the protein surface. When this condition is satisfied (Bottom row), we state a 0.1 Å smaller radius as the biggest probe able to enter, defining the effective pore radius, *r*_E_^17^. This 0.1 Å dissimilarity makes a dramatic difference in the probe’s ability to enter into the inner vestibule (compare the middle with the bottom row).

We measured *r*_E_ in all crystallographic structures available today of four different K^+^-channels: Kv1.2/2.1 paddle-chimera, KvAP, MthK and KcsA channels (Table 1). For Kv 1.2/2.1, SURF, MSMS and HOLLOW gave “average” values of *r*_E_ = 4.5 Å, *r*_E_ = 4.5 Å, and *r*_E_ = 5.2 Å, respectively. For KvAP, *r*_E_ values were less regular but, in general agreed with those of Kv1.2/2.1. Also, for MthK, *r*_E_ = 5.3–5.6 Å, *r*_E_ = 5.3–5.5 Å, and *r*_E_ = 5.7–6.0 Å, for SURF, MSMS, and HOLLOW, respectively. For KcsA *r*_E_ values were much smaller, as expected for a closed channel.

## The Radius of Capture

We compared *r*_E_ with functional estimates of radius of capture (*r*_C_) in the two counterparts of the MthK and Kv1.2/2.1 structures: the BK, and the Shaker channels, respectively^15^,^16^ (To our knowledge, these are the only *r*_C_ estimates available for K^+^-channels). *r*_C_ is an assessment of the dimension of the pore’s entrance obtained from single channel current measurements in a regime in which diffusion of K^+^ ions into the pore entrance is the rate limiting step for passage through the channel. If the transmembrane voltage and/or the recording solution viscosity are high enough, as the applied voltage increases the unitary ionic current departs significantly from the Ohms law; approaching a plateau. This asymptotic saturation amplitude represents the diffusion limited rate with which ions approach the channel entrance. Thus, the bigger the pore entrance, or the higher the ion concentration, or the larger the diffusion coefficient, the higher is the limiting asymptotic current. If the pore is assumed to be a hemispheric sink into which approaching ions vanish, it is possible from diffusional collisions theory to infer its dimension from the limited unitary current, *i*_DL_, according to^15^,^16^,^18^,^19^,^20^.


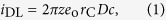


where z is the permeant ion valence, e_o_ the elementary charge, c the bulk ion concentration, and D its diffusion coefficient. If the permeant ion is a point charge, *r*_C_ represents the average radius of the pore’s entrance^15^,^18^. However, because ions have finite size and pores usually aren’t circular, shape and size must be considered. For simplicity, let’s assume that ions are solid spheres reaching a circular pore with radius *r*_O_, then,


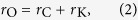


where *r*_K_ is the ion´s spherical radius^15^,^18^,^20^. For point charges, *r*_O_ = *r*_C_. However, for a given, experimentally obtained *r*_C_, estimates of *r*_O_ will grows linearly with *r*_K_, because the number of effective collisions will be indistinguishable from those produced by point charges having identical radial deviations from the central trajectory^16^.

To reach asymptotic currents values at experimentally possible voltages (between 100–350 mV), 2 molar sucrose was added to the cytosolic recording solution to increase viscosity by ~7-fold, and values of *r*_C_ = 2.2 Å and *r*_C_ = 0.8 Å, were obtained for BK and Shaker internal entrances, respectively^15^,^16^. According to Eq. 2, by knowing *r*_K_, we could obtain *r*_O_, their vestibule average radius; nevertheless, the hydrated ion size is not well defined, in part because hydration is a fuzzy arrangement of water molecules bound with dissimilar energies and lifetimes^15^,^16^,^25^.

To compare structural (*r*_E_) with functional (*r*_C_) estimates of the internal pore vestibule, here we make *r*_O_ = *r*_E_, then:


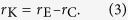


To be meaningful, Eq. 3 must provide consistent estimates for *r*_K_. In fact, Table 1 show that this was true across all structural models and channel types, regardless of the surface calculation algorithm used. Although, HOLLOW gave consistently larger estimates, *r*_K_ fell mostly in the 3.5–4.0 Å interval, providing a robust validation for pore openings dimensions. Strikingly, the values for *r*_K_ are well in agreement with the hydrated size of K^+^ ions estimates obtained using other very different experimental and theoretical methods^25^,^26^,^27^,^28^,^29^. A 3.5–4 Å radius sphere occupies a volume of 180–270 Å^3^ which is comparable to the anti-prismatic geometric arrangement of waters co-crystalized “in flagrante” with K^+^ in the KcsA structure (~300 Å^3^)^4^,^30^, and in agreement with several studies, suggest a coordination number of 8 or less^25^,^26^,^27^,^29^. Of course, our intention is not to provide another estimate of the hydrodynamic size of hydrated K^+^ but to show the robustness of Eq. 3 in comparing structural and functional data.

We should bear in mind that the *r*_C_ estimates are restricted to the hydrated path to the pore entrance, and may contain two sources of error: on the one hand, an overestimation of *r*_C_ arises with the presence of the gating ring in BK and the tetramerization domain in Shaker. In typical recording solutions, both contribute modestly to single channel conductance (up to 30%) by increasing the local K^+^ concentration in corresponding proportion^9^,^31^,^32^. On the other hand, a narrowing of the hydrated path promoted by the hydrophobic nature of the channel’s inner cavity residues may cause an underestimation of the sectional access to the pore; nevertheless at very positive voltages the water content in the cavity matches the cavity’s volume^33^. In this regard, it is interesting to note that our estimates of *r*_E_ for Kv1.2/2.1 chimera are in the 4.4–5.2 Å range, just enough to allow K^+^ hydration to occur in this confined space as in bulk solution, according to molecular dynamics calculations on Shaker^34^. In narrower pores, hydration number decreases and the energetic cost of putting ions inside may be too high to make permeation possible^34^. In contrast, a wider cavity as MthK with *r*_E_ = 5.3–5.9 Å, may provide the incoming hydrated K^+^ with a second, loosely attached hydration shell that may reduce the ion’s energy and/or the friction of its rigid hydration cage^16^,^34^,^35^.

The *r*_C_ estimates shown here stem from studies in liquid phase, at room temperature, away from equilibrium, and with fully operative friction forces^15^,^16^,^20^. By contrast, K^+^ channel structures are obtained at low temperature, in solid phase, and in equilibrium. Thus, the agreement between these functional and structural data is satisfying. They gave consistent estimations of the hydrodynamic radius of K^+^ ions in physiological solutions and, while we cannot ascertain the degree of opening of KvAP structures, we can propose that KcsA structures are closed, whereas Kv1.2/2.1, KvAP, and MthK are *bona fide* open channels having different pore dimensions, possibly explaining differences in unitary conductance^16^.

## Additional Information

**How to cite this article**: Moldenhauer, H. *et al*. Effective pore size and radius of capture for K^+^ ions in K-channels. *Sci. Rep.* **6**, 19893; doi: 10.1038/srep19893 (2016).

## Figures and Tables

**Table 1:** Effective pore radii (*r*_E_) for several K^+^ channel crystals.

**Protein**	**PDB code**	**Crystal conditions & resolution**	**Sphere radius (Å)**						**Ref.**
			**hollow**		**Surf**		**msms**		
			**r**_**E**_	**r**_**K**_	**r**_**E**_	**r**_**K**_	**r**_**E**_	**r**_**K**_	
Kv1.2/2.1 Paddle chimera	2R9R	Complex with lipids at 2.4 Å	5.2	4.4	4.5	3.7	4.4	3.6	7
	4JTA	Complex with ChTx at 2.5 Å	5.2	4.4	4.5	3.7	4.6	3.8	2
KvAP	2A0L	3.9 Å	4.4	3.6	4.1	3.3	4.2	3.4	36
	1ORQ	3.2 Å	4.9	4.1	5.1	4.3	5.5	4.7	37
MthK	4HYO	1.65 Å	5.9	3.7	5.6	3.4	5.5	3.3	10
	3LDC	1.45 Å	5.7	3.5	5.3	3.1	5.3	3.1	38
	1LNQ	3.3 Å***	5.8	3.6	5.6	3.4	5.5	3.3	3
KcsA	1K4C	2.0 Å	2.1		<1.4		<1.4		4
	1BL8	3.2 Å	<1.4		<1.4		<1.4		5

Surface calculation algorithms were used to define the cast envelope left by a spherical probe of varying radius^22^,^23^,^24^. A probe able to enter the pore from the channel’s cytosolic side created a continuous surface connecting the cytosolic face with the pore walls. However, when the probe cannot longer enter, it casts a bump in the place of the pore. Using this criterion, *r*_E_ was defined as the largest sphere able to enter the pore. *r*_K_ was calculated from Eq. (3). For Kv1.2/2.1 and KvAP we used *r*_C_ = 0.8 Å^16^, while *r*_C_ = 2.2 Å was used for MthK channels^15^. ChTx stand for charybdotoxin. ***The biological assembly of this structure is not fully symmetric at the pore entrance.
